# Supplementary material for: Universal atrial coordinates applied to visualisation, registration and construction of patient specific meshes
Source: Med Image Anal. 2019 Jul;55:65–75. doi: 10.1016/j.media.2019.04.004 (PMC6543067; doi:10.1016/j.media.2019.04.004)
Supplement: Supplementary Data S1 — Supplementary Raw Research Data. This is open data under the CC BY license http://creativecommons.org/licenses/by/4.0/ [file mmc1.pdf]

1. Supplementary Material: Universal atrial coordinates applied to visualisation, registration and construction of patient specific meshes

1.1. Assigning atrial regions

In this study, regions were labelled by selecting points at the boundary of the structure and the atrial body and using a geodesic distance criterion, as follows. For the LA, one point was selected per PV and two points for the appendage. For the RA, a point was selected for each of the SVC, IVC and CS, and two points were selected for the appendage. These points are shown in Fig 1, and details for their selection are as follows.

For each of the PVs, the SVC, and the IVC, a point was selected at the boundary of the structure and the atrial body (see Fig 1 A). For the left and right atrial appendages (LAA and RAA), one point was selected on the tip of the appendage, and a second point was selected on the boundary of the appendage and atrial body (see Fig 1 E).

The selected points were then used to define atrial structures using the following methodology. First of all, the boundary edges of the left and right atrial meshes were identified, and separated into lists of connected edges (representing the boundaries of the MV and TV; each of the PVs; the SVC, IVC and CS). For each of the PVs, the SVC and the IVC, geodesic paths were calculated from the nodes in the boundary edge list to the rest of the mesh. Then for each node of the mesh, the minimum distance to the set of nodes in the boundary edge list was calculated (see Fig 1B). Regions were then defined as the set of nodes closer to the nodes in the boundary edge list for that region than the selected point (see Fig 1C).

A similar technique was used to define the left and right atrial appendages. Geodesic distances were calculated from the point at the tip of the appendage to the rest of the mesh (see Fig 1D), and nodes closer to the tip than the point selected at the boundary of the appendage and atrial body, were defined as appendage (see Fig 1E). Geodesic paths were calculated using a marching cubes algorithm (Peyre, 2009; Roney et al., 2015).

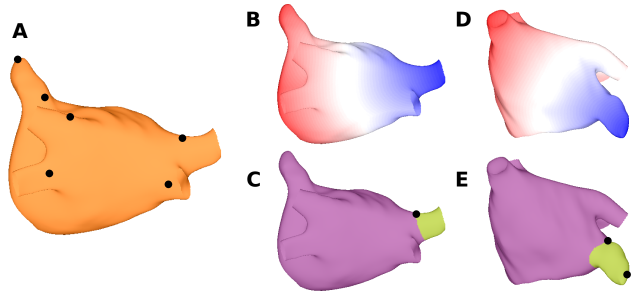

Fig. 1. Assignment of veins and appendages: (A) Example points selected for assignment of each region. (B) Geodesic map for the RSPV. (C) Points with geodesic distance less than the selected point in black are assigned to the RSPV region. (D) Geodesic map for the LAA. (E) Points with geodesic distance less than the selected point at the LAA-LA junction (shown in black) are assigned to the LAA region.

1.1.1. Incorporating additional structures - Bachmann's bundle and the coronary sinus

Bachmann's bundle and the coronary sinus require additional steps for their construction because these structures are not fully determined by the UAC system. Part of the CS structure was assigned to the RA mesh that was generated from MRI data (see Section 1.1) and expressed in UACs, and this was then extended to a cylindrical structure. This was implemented by identifying the rim of boundary nodes of the CS structure on the MRI mesh, projecting these to a plane, and calculating a center line trajectory for the CS cylinder. This trajectory was constructed by selecting a target end point on the LA mesh, finding a geodesic path from this point to the point on the LA closest to the CS boundary nodes and then projecting this path off of the mesh. A spline was then constructed to join the distal nodes of the path to a trajectory from the centre point of the boundary rim that is tangential to the normal of the boundary plane. The resulting path is then in the tangential direction to the initial CS structure at the start of the cylinder, and follows the morphology of the LA mesh. The boundary nodes were then marched forward by shifting the boundary plane along the tangent to the centre line trajectory, for both this arrangement, and for nodes at the mid-points, in order to create triangular elements. To introduce a degree of taper, the diameter of the bounding circle of the nodes was decreased along the length of the cylinder. This is demonstrated in Supplementary Fig 2 A.

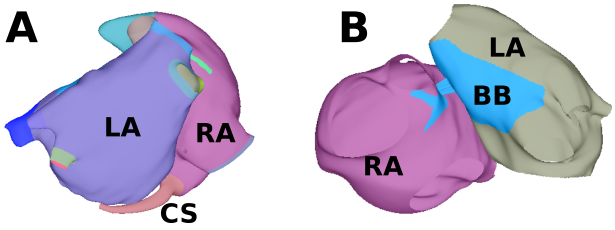

Fig. 2. Including the coronary sinus and Bachmann's bundle in the model: (A) The inclusion of the CS. (B) The inclusion of BB.

LA and RA components of BB were initially assigned using scalar mapping as for the other structures. It is not possible to assign the component of BB between the LA and RA using UACs because this region is not close enough to either mesh. Instead, the LA and RA components of BB were joined to each other using an automatic mesh construction technique. An equal number of connected nodes along the boundary of each component were selected and then new nodes were distributed at an equal spacing along the vector joining equivalent nodes on each component, and finally nodes were added at midpoints to construct triangular elements. This is demonstrated in Supplementary Fig 2 B.

1.1.2. Connections

To construct a bilayer model, discrete resistance line connections were added between each vertex of the LA endocardial mesh and the closest LA epicardial vertex. Connections between the sinus node (SAN) and RA were assigned to the model by mapping the original mesh SAN line connection vertices to the new mesh using UACs. The LA and RA FO rims were

electrically connected by joining the closest LA and RA nodes using line connections. In addition, the half of the distal CS rim closest to the LA body was joined to the closest LA nodes using line connections.

1.1.3. Sensitivity to choice of markers

For the geometry shown in Fig 3, it was difficult to segment the LSPV and LIPV structures. The effects of moving the LSPV and RSPV markers selected for assigning the roof boundary to the base of the veins is shown in Fig 3. The median angle difference compared to the baseline UAC selection was 8.1°.

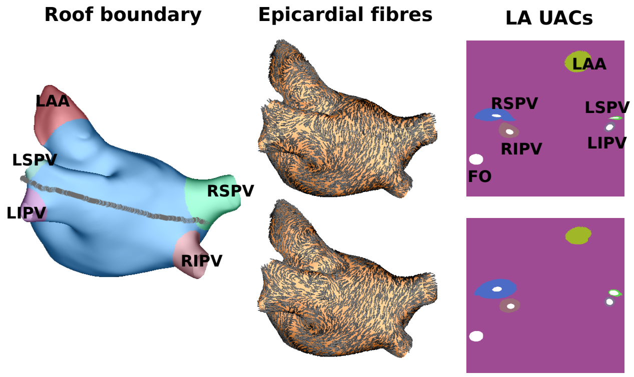

Fig. 3. Point sensitivity. The roof boundary nodes for the Laplace solve was moved to the base of the veins. The epicardial fibres and LA UACs are shown for the original boundary selection (top) and modified boundary selection (bottom).

References

Peyre, G., 2009. Toolbox fast marching. MATLAB Central File Exchange Select 2.

Roney, C.H., Tzortzis, K.N., Cantwell, C.D., Qureshi, N.A., Ali, R.L., Lim, P.B., Siggers, J.H., Ng, F.S., Peters, N.S., 2015. A technique for visualising three-dimensional left atrial cardiac activation data in two dimensions with minimal distance distortion, in: Engineering in Medicine and Biology Society (EMBC), 2015 37th Annual International Conference of the IEEE, IEEE. pp. 7296–7299.
